# Evaluation of onchocerciasis seroprevalence in Bioko Island (Equatorial Guinea) after years of disease control programmes

**DOI:** 10.1186/s13071-016-1779-8

**Published:** 2016-09-20

**Authors:** Ana Hernández-González, Laura Moya, María J. Perteguer, Zaida Herrador, Rufino Nguema, Justino Nguema, Pilar Aparicio, Agustín Benito, Teresa Gárate

**Affiliations:** 1 Helminth Unit, Parasitology Department, Centro Nacional de Microbiología, Instituto de Salud Carlos III, Crtra. Majadahonda-Pozuelo, km 2.2, 28220 Majadahonda, Madrid Spain; 2 National Centre for Tropical Medicine, Instituto de Salud Carlos III, C/ Sinesio Delgado, 4, 28029 Madrid, Spain; 3 Jimenez Diaz Foundation University Hospital, Avda. Reyes Católicos, 2, 28040 Madrid, Spain; 4 Network Biomedical Research on Tropical Diseases (RICET), Madrid, Spain; 5 National Program for Control of Onchocerciasis and other Filariasis, Ministry of Health, Malabo, Equatorial Guinea

**Keywords:** Onchocerciasis, *Onchocerca volvulus*, Bioko Island, Equatorial Guinea, Seroprevalence, Ov-16/IgG4 ELISA

## Abstract

**Background:**

Onchocerciasis or “river blindness” is a chronic parasitic disease caused by the filarial worm *Onchocerca volvulus*, transmitted through infected blackflies (*Simulium* spp.). Bioko Island (Equatorial Guinea) used to show a high endemicity for onchocerciasis. During the last years, the disease control programmes using different larvicides and ivermectin administration have considerably reduced the prevalence and intensity of infection. Based on this new epidemiological scenario, in the present work we aimed to assess the impact of the strategies applied against onchocerciasis in Bioko Island by an evaluation of IgG4 antibodies specific for recombinant Ov-16 in ELISA.

**Methods:**

A cross-sectional study was conducted in Bioko Island from mid-January to mid-February, 2014. Twenty communities were randomly selected from rural and urban settings. A total of 140 households were chosen. In every selected household, all individuals aged 5 years and above were recruited; 544 study participants agreed to be part of this work. No previous data on onchocerciasis seroprevalence in the selected communities were available. Blood samples were collected and used in an “ELISA in-house” prepared with recombinant Ov-16, expressed and further purified. IgG4 antibodies specific for recombinant Ov-16 were evaluated by ELISA in all of the participants.

**Results:**

Based on the Ov-16 ELISA, the onchocerciasis seroprevalence was 7.9 %, mainly concentrated in rural settings; samples from community Catedral Ela Nguema (# 16) were missed during the field work. Among the rural setups, communities Inasa Maule (# 7), Ruiché (# 20) and Barrios Adyacentes Riaba (# 14), had the highest seropositivity percentages (29.2, 26.9 and 23.8 %, respectively). With respect to the urban settings, we did not find any positive case in communities Manzana Casa Bola (# 3), Colas Sesgas (# 6), Getesa (# 8), Moka Bioko (# 9), Impecsa (# 10), Baney Zona Baja (# 12) and Santo Tomás de Aquino (# 1). No onchocerciasis seropositive samples were found in 10-year-old individuals or younger. The IgG4 positive titles increased in older participants.

**Conclusions:**

A significant decline in onchocerciasis prevalence was observed in Bioko Island after years of disease-vector control and CDTI strategy. The seroprevalence increased with age, mainly in rural settings that could be due to previous exposure of population to the filarial parasite, eliminated by the control programmes introduced against onchocerciasis. A new Ov-16 serological evaluation with a larger sample size of children below 10 years of age is required to demonstrate the interruption of transmission of *O. volvulus* in the human population of Bioko Island (Equatorial Guinea) according to the WHO criteria.

## Background

Onchocerciasis or “river blindness” is a chronic parasitic disease caused by the filarial worm *Onchocerca volvulus*. It is transmitted through the repeated bites of infected blackflies (*Simulium* spp.) from human to human. In the human body, the larvae form nodules in the subcutaneous tissue, where they mature to adult worms. After mating, the female adult worm release microfilariae (MF) that move through the body. When MF die they cause a variety of pathological conditions, affecting principally eyes and skin [1].

In 1974, the onchocerciasis control programme (OCP) was launched in the African continent, with the aim of eliminating the vector blackfly by regular aerial larviciding. This strategy was encouraged by the decision taken by Merck in 1987 to make ivermectin (Mectizan) available free of charge through the Mectizan Donation Programme (MDP) [2]. Later on, in 1995, the African Programme for Onchocerciais Control (APOC) begun annual administration of the community-directed treatment with ivermectin (CDTI) [3, 4]. APOC based the drug administration on the previous assessment of the local disease endemicity by rapid epidemiological mapping of onchocerciasis (REMO) [5]. All these interventions have demonstrated efficacy in controlling onchocerciasis as a public health problem in Africa [6].

Bioko Island, located in Equatorial Guinea, used to show a high endemicity for onchocerciasis [7, 8]. The introduction of the disease control programmes, mentioned above, allowed the reduction in the prevalence and intensity of infection [9], and the presumptive elimination of the onchocerciasis vector, the Bioko form of *Simulium yahense* [10]. The CDTI has been continued until now. At this stage, surveillance and evaluation activities are especially necessary to verify that the parasite transmission has been interrupted [11]. Onchocerciasis detection has been traditionally carried out by nodule palpation and microscopic examination of MF in skin snips, although nodule palpation has poor diagnostic precision and morphological MF identification requires substantial expertise, is time-consuming and shows poor sensitivity [12]. In locations where onchocerciasis prevalence has dropped down thanks to CDTI, and disease elimination needs to be evaluated, new survey and diagnostic tools are required. Therefore, innovative strategies for onchocerciasis accurate diagnosis, immunological and DNA-molecular approaches, have been developed during the last decades [13–15].

Within immunodiagnosis options, the evaluation of IgG4 responses to the Ov-16 antigen, by ELISA and/or rapid-format card test, has emerged as an alternative tool to determine the impact of the disease elimination progress [16, 17]. *Onchocerca volvulus*-Ov-16 antigen belongs to the phosphatidylethanolamine-binding protein family (A.N.: P31729 [18, 19]), and expressed as a recombinant antigen allowed the early and specific detection of new *Onchocerca* infections in children, with good sensitivity [17]. WHO has been using the Ov-16/IgG4 system to verify the interruption of transmission of human onchocerciasis, besides ophthalmological and/or entomological assessments, since 2001 [20]. This serological approach has been employed, among others, to certify the transmission suppression of onchocerciasis in the Americas [21–23] and in some African endemic regions [4, 24, 25], and again it has been recently recommended by WHO in the last onchocerciasis guidelines [26].

The present work represents a preliminary study, which aimed to assess the impact of CDTI strategy on onchocerciasis control in Bioko Island by the evaluation of IgG4 antibodies specific for recombinant Ov-16 by using ELISA [20].

## Methods

### Study area

The study took place in Bioko Island from mid-January to mid-February, 2014. The Island is a part of the Republic of Equatorial Guinea, which also includes Rio Muni on the mainland and the island of Annobon. Bioko Island comprises two zones: Bioko Norte (Malabo, Baney and Rebola districts) and Bioko Sur (Luba and Riaba districts). It is located in the Bay of Guinea in Central Africa, about 40 km southwest of the Cameroon coast. The surface area of Bioko Island is of approximately 2017 km^2^ and it is about 72 km in length. Most of the 260,000 inhabitants live in the capital, Malabo.

The island has a humid tropical climate. Mean daily maximum and minimum temperatures range between 29–32 °C and 19–22 °C, respectively. The rainfall is highly seasonal, with a dry season which lasts from December to April; many of the rivers and streams dry up soon after the end of the rainy season. Before the elimination of the vector blackfly, the levels of vector biting were lowest during January to April coinciding with the dry season reduction in suitable breeding sites [27]. Currently, seasonality and rainfall are not expected to influence the transmission of *O. volvulus*, as there is no evidence of vector re-emergence in the island since 2005.

### Study design, sample size and sampling technique

A cross-sectional study was conducted. Sampling was carried out by multistage cluster survey. The sample size was computed using Epi-Info version 3.4.1 free software considering the following parameters: hypothesized prevalence of 10 % and a standard error of 2 %. We assumed a design effect of 2, corresponding to the complex design. The initial sample size was 450. It was increased (+20 %) in prevision of missing data. The equation used is included below: *n* = DEFF p (1-p)/e^2, where DEFF is the design effect, e is the desired standard error and p is the prevalence.

**Fig. 1:**
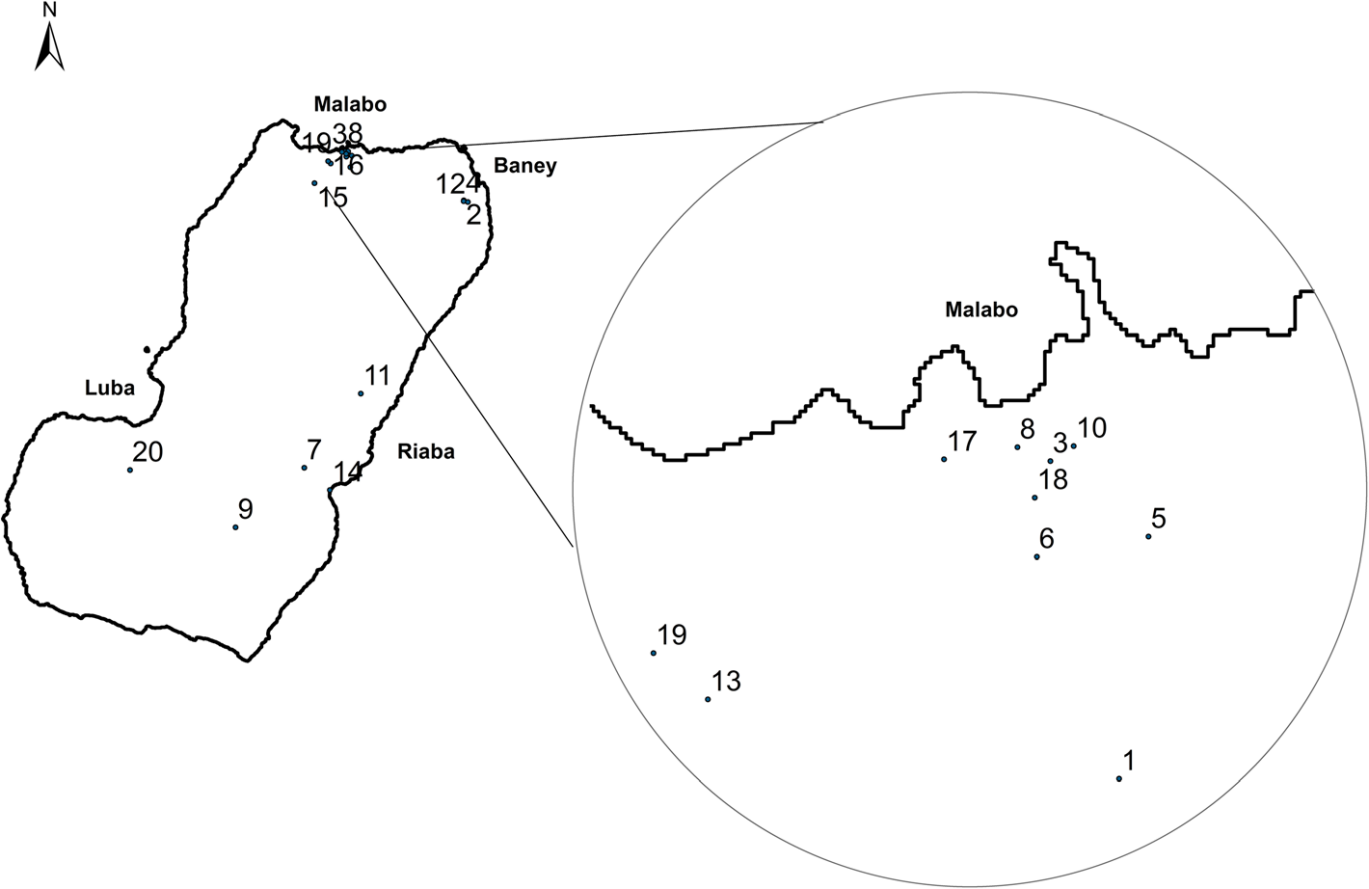
Distribution of the randomly selected community clusters, Bioko Island, Equatorial Guinea: #1, Santo Tomás de Aquino; #2, Baney Zona Media B1; #3, Manzana Casa Bola; #4, Zona Alta A1 Baney; #5, Alcalde 1 Malabo; #6, Colas Sesgas; #7, Inasa Maule; #8, Getesa; #9, Moka Bioko; #10, Impecsa; #11, Bilelipa; #12, Baney Zona Baja; #13, Santa Maria 4B Malabo; #14, Barrios Adyacentes Riaba; #15, Sampaka 1 Malabo; #17, Zona “D” C/N° 1–25 Malabo; #18, Cachirulo; #19, Santa María 4A; #20, Ruiché

First, 20 communities were randomly selected with probability proportional to size (Fig. 1). Secondly, sampling units were randomly selected households from an updated census from each community, provided by the head of the village (in rural areas) or neighbourhood (in urban zones). In every selected household, all individuals over 5 years of age who had permanently lived in Bioko Island during the last 5 years were recruited. People who were born outside Equatorial Guinea were excluded.

### Data collection

A closed-ended structured questionnaire was administered to every study participant by trained medical personnel. The questionnaire comprised socio-demographic and clinical information and was pre-tested on close areas not included in this study for clarity and cultural acceptability. Each interview was made by house-to-house visit. If the study participant was less than 15 years of age, the questionnaire was answered by a parent or guardian. Those children under 5 years of age, no Equatoguinean nationality, and/or who had changed residence in the last 5 years were excluded.

The coordinates (longitude and latitude) of every selected household were also recorded on-site using Dakota™10 Garmin single handheld GPS receivers.

### Skin-snips

Three skin snips specimens were collected from every participant (two from right iliac crest, one from left iliac crest). No special collection time was considered as the microfilariae of *O. volvulus* are non-periodic. Two samples were immersed in normal saline solution to prevent the preparation from drying out and sent to the local hospital laboratory to be read under a microscope (magnification of 100×) after 24 h. Results were expressed for each individual as ‘positive’ or ‘negative’. Laboratory results were recorded on the original (field) registration form.

### Blood samples

Blood drops were taken from peripheral blood samples that were collected from each individual [28] and placed on a label circle of Whatman No. 2 filter paper (Milipore). Filter paper blood samples were dried, individually wrapped in plastic bags with a silica desiccant and stored in coolers at 4 °C until they could be transferred to a refrigerator, where they were stored until further analysis.

### Production of Ov-16 recombinant antigen

The Ov-16 nucleotide sequence [18], without the signal peptide, was amplified by PCR from a recombinant pMAL-Ov-16 plasmid, kindly donated by Professor JE Bradley, School of Life Sciences and University of Nottingham, UK. A poly-His tail was added to the carboxy-terminus of the amplified Ov-16 sequence, which was then subcloned into pGEX-6P-1 plasmid (GE Healthcare, Little Chalfont, UK), a glutathione S-transferase fusion vector. The GST-Ov-16 recombinant protein was purified by Glutathione Sepharose 4B affinity chromatography (GE Healthcare) according to the manufacturer’s recommendations. Later, separation of the recombinant protein from the glutathione S-transferase moiety was accomplished by site-specific proteolysis using PreScission^TM^ Protease reaction (GE Healthcare). After dialyzing against PBS, the Ov-16-His recombinant protein was repurified by Ni^2+^ Sepharose 4B affinity chromatography (GE Healthcare) according to the manufacturer’s recommendations. Finally, the Ov-16-His was again dialyzed against PBS and quantified with Pierce™ BCA Protein Assay Kit (Thermo Scientific, Rockford, IL, USA).

### Serological assessment

#### In-house ELISA test

In the laboratory, serum samples were eluted from Whatman filter paper as follows. Four 3 mm-punches of blood saturated filter paper were placed in 200 μl of dilution buffer (phosphate-buffered saline (PBS)-Tween 20 0.05 %-skim milk 3 %) and eluted overnight at 4 °C, according to Lindblade et al. [29] with small modifications.

The serum elution was run in duplicate in a standard enzyme-linked immunosorbent assay (ELISA) to detect IgG4 antibodies against the Ov-16 recombinant antigen essentially as previously described [25]. Briefly, 96-well polystyrene plates (Nunc MaxiSorp® flat-bottom 96 well plate) were sensitized with the purified Ov-16 recombinant protein (0.5 μg/ml; 100 μl/well) in carbonate buffer pH 9.6, overnight at 4 °C. Later, the plates were washed three times with phosphate PBS, pH 7.4 plus 0.05 % Tween 20 (PBST, washing buffer) and blocked in dilution buffer (PBST plus 0.3 % skim milk) for 1 h at 37 °C. After blocking, plates were washed 3 times as described above. The diluted serum samples (100 μl) were added in duplicate to the corresponding wells; eluted positive and negative controls, equally processed and diluted, were also introduced. After 1 h of incubation at 37 °C, the plates were PBST-washed 5 times and bound antibodies were detected by exposure to horseradish peroxidase conjugated mouse anti-human IgG4 (Southern Biotech, Birmingham, AL, USA), 1/4000 in PBST; the plates were incubated and washed as described above. The substrate buffer was prepared with an ABTS tablet (2,2′-Azino-bis (3-ethylbenzothiazoline-6-sulfonic acid) diammonium salt; Sigma-Aldrich, A9941, Saint Louis, MO, USA) plus 20 ml of phosphate-citrate buffer with sodium perborate (Sigma-Aldrich P4922); 100 μl of substrate buffer was added to each well and then the plate was incubated at 37 °C in darkness. The plates were read at 405 nm with a 620 nm reference filter when the 1/8 positive control dilution was around 0.5 OD (see below).

We used a standard curve on each plate to identify positive samples and permit comparisons between plates and over days, as previously described [25, 29]. The standard curve was prepared using a positive onchocerciasis sample, obtained from an onchocerciasis patient confirmed by both clinical manifestations and parasitological diagnosis; the standard curve dilutions (1/8; 1/16; 1/32; 1/64; 1/128) were prepared through elution of the blood sample placed on a Whatman filter paper. Each of these dilutions was assigned the arbitrary units shown in Table 1. The cut-off was determined after analyzing 83 negative samples from sub-Saharan (*n* = 33) /Spanish/Latin American individuals and 5 positive samples (parasitologically and clinically confirmed as *O. volvulus*-positive individuals). The cut-off was set at 40 arbitrary units corresponding to the 1/64 dilution by identifying the value that optimized both sensitivity and specificity.

**Table 1:** Standard curve dilutions on each Ov-16-IgG4 ELISA plate. The mean and standard deviation obtained for each dilution and the arbitrary units assigned to each dilution are shown. The plates were read when the 1/8 dilution reached a 0.5 OD value. The 1/64 dilution was determined as the cut-off, hence samples with OD-values above this value were considered positive

Standard curve dilutions	Target mean (OD)	Standard deviation	Units
1/8	0.512	0.058	320
1/16	0.243	0.032	160
1/32	0.119	0.020	80
**1/64**	**0.061**	0.010	40
1/128	0.032	0.005	20

The cut-off dilution and the corresponding OD value are marked in bold

#### Commercial test

Positive onchocerciasis samples by ELISA-Ov-16/IgG4 were re-tested in a rapid format for the detection of IgG4 antibodies against Ov-16 recombinant antigen (SD Onchocerciasis IgG4-Bioline, Standard Diagnostics, Inc., Gyeonggi, South Korea); the procedure shows a sensitivity of 81.1 % and a specificity of 99.0 % according to the manufacturer. The immunochromatographic tests were developed following the manufacturer’s recommendations, using 10 μl blood samples. We considered a sample as positive when it was possible to see a red line on the test line even when the line was very weak.

### Data analysis

The collected data were double entered into a data entry file using EpiData software, V.3.1. Frequencies, means and standard deviations (SD) were computed to summarize the data. Antibody prevalence was defined as the proportion of positive results among subjects who had definite results for the Ov-16 ELISA. Bivariate analyses by age group were performed with *χ*^2^ test for categorical data. Where a cell value was below 5, Fisher’s exact test for two-way tables was applied. The criterion for significance was set at *P* < 0.05 based on a two-sided test. All statistical analyses were performed using SPSS v.22 (SPSS Inc., Chicago, Illinois, USA).

## Results and discussion

A total of 140 houses and 544 study participants agreed to be part of this study. None of the 544 skin snip assessments for MF detection was found positive (data not shown). Blood samples on Whatman paper were collected and analyzed from 531 participants, as the samples from community # 16 were missed during the field work.

Based on the Ov-16 ELISA, the data obtained in the study of the 19 communities showed that the onchocerciasis seroprevalence was 7.9 %, mainly concentrated in rural settings (Table 2). To confirm this result, the positive samples were checked by a commercial test that yielded similar data (40 were positive, 1 was undetermined and 1 negative).

**Table 2:** Number (percent) of positive samples in Ov16/IgG4-ELISA regarding community type. Statistically significant differences were found between rural and urban settings: Chi-square test: *χ* ^2^ = 13.25, *df* = 2, *P* < 0.001

Setting	Positive samples (n)	Percent positive samples	95 % CI
Rural (*n* = 219)	29	13.2	9.4–18.4
Urban (*n* = 312)	14	4.5	2.7–7.4

Table 3 shows the distribution of the onchocerciasis-seropositive individuals in the different surveyed communities. As indicated above, rural communities exhibited the highest number of seropositive individuals for onchocerciasis, with significant differences in relation to the urban ones. Among the rural setups, communities # 7, # 20 and # 14, had the highest seropositivity percentage (29.2, 26.9 and 23.8 %, respectively). The higher prevalence detected in rural communities emphasizes the problem of control strategies implementation in this kind of setting due to difficulties in access, cultural barriers and preference for traditional methods which has already been observed before [30]. With respect to the urban settings, we did not find any positive case in communities # 3, # 6, # 8, # 9, # 10, # 12 and # 17, although communities # 2 and # 18 showed 15.2 and 14.3 % of seropositivity, respectively.

**Table 3:** OV16-IgG4 seropositivity distribution in the surveyed communities. Total number of individuals checked in each community, the number of positive, negative and dubious samples in each community (*n*) and the percent (%) of positivity, negativity and dubious results in each community are shown

	Community	Type of setting	Total	NEG		UND		POS	
				*n*	%	*n*	%	*n*	%
1	Santo Tomás de Aquino	Urban	12	11	91.7	0	0	1	8.3
2	Baney Zona Media B1	Rural	33	27	81.8	1	3.0	5	15.2
3	Manzana Casa Bola	Urban	26	26	92.9	0	0	0	0
4	Zona Alta A1 Baney	Rural	35	31	86.1	1	2.9	3	8.6
5	Alcalde 1 Malabo	Urban	27	24	85.7	0	0	3	11.1
6	Colas Sesgas	Urban	31	31	100.0	0	0	0	0
7	Inasa Maule	Rural	24	16	66.7	1	4.2	7	29.2
8	Getesa	Urban	30	29	90.6	1	3.3	0	0
9	Moka Bioko	Rural	21	19	90.5	2	9.5	0	0
10	Impecsa	Urban	30	27	90.0	3	10.0	0	0
11	Bilelipa	Rural	25	21	80.8	2	8.0	2	8.0
12	Baney Zona Baja	Rural	34	32	94.1	2	5.9	0	0
13	Santa Maria 4B Malabo	Urban	39	37	92.5	1	2.6	1	2.6
14	Barrios Adyacentes Riaba	Rural	21	16	72.2	0	0	5	23.8
15	Sampaka 1 Malabo	Urban	38	33	86.8	1	2.6	4	10.5
17	Zona “D” C/N° 1–25 Malabo	Urban	17	17	94.4	0	0	0	0
18	Cachirulo	Urban	21	18	85.7	0	0	3	14.3
19	Santa María 4A	Urban	41	39	86.7	0	0	2	4.9
20	Ruiché	Rural	26	19	73.1	0	0	7	26.9

*Abbreviations*: *ID* identification number, *NEG* negative results, *POS* positive results, *UND* undetermined results

**Fig. 2:**
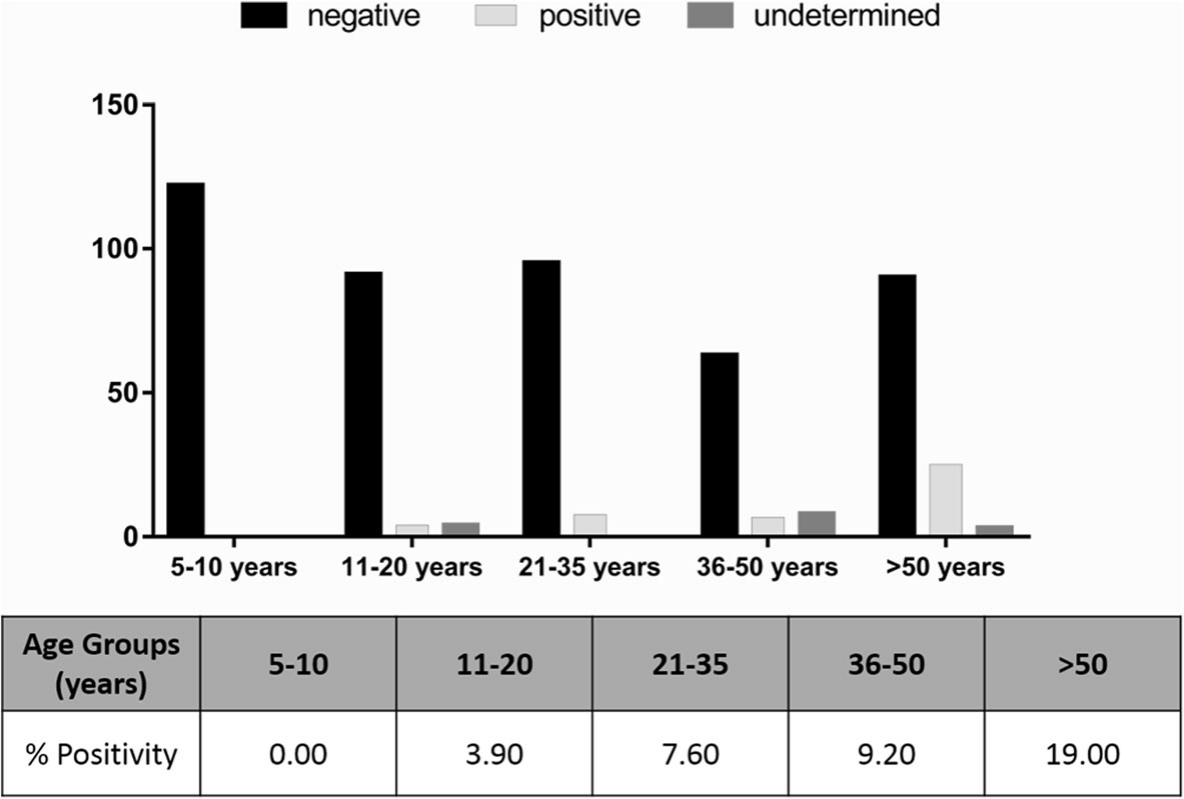
Onchocerciasis seropositivity for age groups. Y axis, number of samples. Statistically significant differences were found between percentage of positivity in age groups by Chi-square test (*χ* ^2^ = 33.762, *df* = 4, *P* < 0.001). Note: There is a positive sample that was not included because the person's age was unknown

The seroprevalence by age group was also assessed. No positive samples were found in 10 year-old individuals or younger, while participants older than 50 years were the ones with the highest prevalence (*χ*2 = 33.762, df = 4, *P* < 0.001), being 19 % onchocerciasis positive by Ov-16-ELISA (Fig. 2). The percentage of individuals with an undetermined serology increased with age (Fig. 2). A sample was considered undetermined when its absorbance was on the pre-defined cut-off, 40 arbitrary units (Table 1). These undetermined results could correspond to individuals previously in contact with the filaria, which after effective treatment with ivermectin, were losing antibodies to Ov-16 to finally become seronegative.

In this paper we evaluated serologically (Ov-16-ELISA) the impact of control programmes on onchocerciasis carried out in Bioko Island, Equatorial Guinea. We observed a significant decrease in the disease prevalence after years of ivermectin administration; the decline (IgG4 antibody title) was less pronounced in rural communities and individuals aged 10 years and above. Previously published data by Mas et al. [9] showed a reduction in the prevalence and intensity in *O. volvulus* after 8 years of CDTI on Bioko Island; the authors selected rural communities, examined more than 1000 individuals by MF counts in skin-snips, and found out that the disease prevalence had decreased from 74.5 to 38.4 % in the period 1989–1998. The ivermectin distribution continued over time thanks to APOC, reaching coverages between 50 and 75 % in most endemic areas [31]. In parallel, the demonstration of the elimination of the Bioko form *S. yahense* (larviciding with temephos, 2001–2005) [10] promoted a new epidemiological scenario with a sharp decline in parasite load. In such epidemiological situation, more sensitive and specific diagnosis tools, as genomic procedures (PCR) and/or recombinant antigens-serology would be needed in order to evaluate the potential interruption of filarial transmission in Bioko Island. The Ov-16 serology with IgG4-system [18, 19, 29] has been a major advance in the fight against onchocerciasis. It has been widely used for certification of the elimination of the disease in the Americas [32] and in some African regions [4, 24, 25]. The Ov-16 antigen exhibits an excellent performance for onchocerciasis diagnosis, although some cross-reactions with *Mansonella ozzardi* have been reported [19, 33]. *Mansonella ozzardi* is not present in Africa, so the handicap previously referred is not relevant to the present study. No such cross-reactivity with the other *Mansonella* spp. has been reported [16, 19]. Nevertheless, it an in-depth analysis of this aspect would be desirable considering that *Mansonella perstans* and *Mansonella streptocerca* are co-endemic with *O. volvulus* and highly prevalent in Africa [34]. With respect to sensitivity, Ov-16-IgG4 assay works as an early and specific marker for *O. volvulus* infection [19]. Interestingly, in the present work only positive IgG4 levels were detected in approximately 7.9 % of the tested population, in individuals aged 11 years and above and mainly settled in rural areas. The prevalence determined was significantly lower than that reported by Mas et al. [9]. Nevertheless, it should be taken into account that Mas and co-workers used MF count that is much less sensitive than immunodiagnosis. Moreover, ivermectin administration has been continued over the last years.

In addition, it should be emphasized that according to the age, the younger population (5–10 year-old) did not present positive serology, meaning that no IgG4 antibodies were detected. This result is extremely important, in view of the fact that the absence of specific IgG4 antibodies highlights no parasite infection in children of Bioko Island, and therefore points to the possible interruption of parasite transmission in the region. Regarding the serologically positive individuals, it should be considered that according to Weil et al. [16], the seropositivity obtained in treated patients could be due to a progressive decline of IgG4 antibodies to Ov-16 over a period of years after apparent onchocerciasis curing. In this sense, the study carried out by Traoré et al. [10] showed the presumptive elimination of the vector of *O. volvulus* in the island since 2005. These results support the interruption of parasite transmission and human infection. Nevertheless, new entomological assessments to verify *S. yahense* elimination are desirable, including the study of other potential vectors which can become infected by the parasite and thus replace the old vector [35].

Our study has some limitations. First, the possibility of cross-reaction of Ov-16 ELISA test with some of *Mansonella* spp., highly prevalent in Africa, should be assessed in the future. Secondly, like other serological tests, the Ov-16 ELISA cannot differentiate recent from historical exposure. Moreover, it is currently not known how long the IgG4 antibody response to the Ov-16 antigen persists. Therefore, a larger number of samples would be necessary in order to define a more accurate seroprevalence in Bioko Island. Also, it would be required to increase the number of samples from children in each community to assess the possible interruption of transmission, since this will allow us to detect possible hot spots.

## Conclusions

In the present work, a decline in onchocerciasis prevalence in humans was determined in Bioko Island after years of vector control measures and CDTI strategy. Nevertheless, the sample population was not large enough and only children above 5 years of age were tested. In the age group 5–10 year-old, none individual had IgG4 antibodies against recombinant Ov-16. Moreover, the Ov-16 seroprevalence increased with age, especially in rural settings, and no *O. volvulus* microfilariae were observed in skin-snips.

Further serological studies with larger human samples are needed, especially in children younger than 10 years, as recommended by recent updated WHO criteria (2016). Besides Ov-16 serology, the verification of *S. yahense* elimination in Bioko Island and the absence of parasite DNA in both human skin-snips and other potential simulid vectors are recommended to confirm the interruption of onchocerciasis transmission in the Bioko Island.

## Abbreviations

ABST 2: 2′-Azino-bis(3-ethylbenzothiazoline-6-sulfonic acid) diammonium salt
APOC: African programme for onchocerciasis control
CDTI: Community-directed treatment with ivermectin
ELISA: Enzyme linked immuno-sorbent assay
GST: Gluthatione-S-transferase
GST-Ov16: *Onchocerca volvulus* Ov-16 recombinant antigen with glutathione S-transferase (GST) tail
IgG4: Immuno-globulin G, subclass G4
MDP: Mectizan donation programme
MF: Microfilariae
MINSABS: Ministry of Health in Equatorial Guinea and Social Welfare
Ov16: *Onchocerca volvulus* recombinant 16-antigen
OCP: Onchocerciasis control programme
OD: Optical density
Ov16-His: *Onchocerca volvulus* Ov-16 recombinant antigen with poly-histidine (His) tail
PBS: Phosphate-buffered saline
PBST: PBS plus 0.05 % Tween 20
REMO: Rapid epidemiological mapping of onchocerciasis
pGEX-6P-1: GST-tagged expression vector. It permits purification on Glutathione Sepharose and simultaneous site-specific cleavage of the recombinant protein
pMAL: Maltose binding protein (MBP)-tagged expression vector. It permits the amylose resin-purification and cleaving with specific protease of the recombinant protein
WHO: World Health Organization
